# Pragmatic AI-augmentation in mental healthcare: Key technologies, potential benefits, and real-world challenges and solutions for frontline clinicians

**DOI:** 10.3389/fpsyt.2022.990370

**Published:** 2022-09-06

**Authors:** Katherine C. Kellogg, Shiri Sadeh-Sharvit

**Affiliations:** ^1^Department of Work and Organization Studies, MIT Sloan School of Management, Cambridge, MA, United States; ^2^Eleos Health, Cambridge, MA, United States; ^3^Center for M^2^Health, Palo Alto University, Palo Alto, CA, United States

**Keywords:** artificial intelligence, AI-augmentation, automation technologies, clinical practice, decision support technologies, engagement technologies, mental healthcare

## Abstract

The integration of artificial intelligence (AI) technologies into mental health holds the promise of increasing patient access, engagement, and quality of care, and of improving clinician quality of work life. However, to date, studies of AI technologies in mental health have focused primarily on challenges that policymakers, clinical leaders, and data and computer scientists face, rather than on challenges that frontline mental health clinicians are likely to face as they attempt to integrate AI-based technologies into their everyday clinical practice. In this Perspective, we describe a framework for “pragmatic AI-augmentation” that addresses these issues by describing three categories of emerging AI-based mental health technologies which frontline clinicians can leverage in their clinical practice—automation, engagement, and clinical decision support technologies. We elaborate the potential benefits offered by these technologies, the likely day-to-day challenges they may raise for mental health clinicians, and some solutions that clinical leaders and technology developers can use to address these challenges, based on emerging experience with the integration of AI technologies into clinician daily practice in other healthcare disciplines.

## Introduction

Artificial intelligence (AI) technologies—computer systems that perform human-like physical and cognitive tasks such as sensing, perceiving, problem solving, and learning subtle patterns of language and behavior (1)—may both enable mental health (MH) clinicians to focus on the human aspects of medicine that can only be achieved through the clinician–patient relationship, and at the same time make therapies more effective. In the health domain, the automation, engagement, and decisions empowered by AI are commonly reviewed by domain experts before they can be implemented in a patient's treatment plan, a process called “augmented intelligence” or “intelligence amplification.” By utilizing the capabilities of AI systems to support clinicians and patients, they transform mental healthcare (MHC) through improving patient experience and retention and the work life of healthcare professionals, and reducing costs (2). AI-Augmentation incorporates a pragmatic approach, led by the frontline clinician, wherein AI technology informs and augments, rather than replaces, clinician experience and cognition (3).

To date, much of the literature on implementing AI technologies in MHC has highlighted the potential clinical and economic value of AI (1), as well as the technical, ethical, and regulatory challenges associated with its effective implementation (4). A research agenda and initiatives for addressing these challenges has also been proposed, such as redesigning elements of the technical and regulatory infrastructure of organizations (4), using robust data and science practices in developing AI technologies (5, 6), studying AI efficacy and fairness (7), and providing formal training to medical professionals (7).

To add to this emerging understanding of effective AI development and use in MHC, we provide a complementary perspective of “Pragmatic AI-Augmentation.” We argue that, in order to realize the promise of AI technologies in MHC, it is necessary to answer three questions: (1) What are some specific AI technologies that frontline MH clinicians can leverage to inform and augment their human intelligence in clinical practice?; (2) What challenges are likely to arise for MH clinicians as they attempt to use these AI technologies in their daily work?; and (3) What solutions can clinical leaders and technology developers use to help address MH clinicians' AI implementation challenges?

Three categories of AI technologies that are of particular interest to MH clinicians are automation technologies, engagement technologies, and clinical decision support technologies (8, 9). AI-based automation technologies allow for the automation of healthcare management processes to optimize healthcare delivery or reduce administrative cost, often via computer vision and machine learning (ML) systems. AI-based engagement technologies allow for engagement with patients using natural language processing (NLP) chatbots and intelligent agents. And AI-based clinical decision-making support technologies allow for early detection and diagnosis through extensive analysis of structured data, most often using ML algorithms and neural networks. While we do not cover all types of technologies in this paper, we provide a framework for patients, clinicians, and stakeholders to think about AI-augmentation in their practice (10). Table 1 illustrates key characteristics, applications, and potential benefits of AI for MH clinicians in each of these three domains. Table 2 details related challenges and solutions required for pragmatic AI-augmentation.

**Table 1:** Key AI technologies, example applications in mental healthcare, and potential benefits.

	**Automation technologies**	**Patient engagement technologies**	**Clinical decision support technologies**
Key AI technologies	• Computer vision •Machine learning • Natural language processing	• Conversational Agents • Chatbots	• Machine learning • Neural networks • Deep learning
Example applications in MH	• Screening for autism, eating disorders • Administering digital surveys • Generating insights from a patient's talking patterns, tone, word choice	• Coaching for smoking cessation, exercise, nutrition • Scripted CBT, MI, dialectical behavioral therapy	• Depression and anxiety prediction • Stroke prediction • Suicide ideation prediction
Potential benefits	• Increase early screening • Increase patient access • Improve clinician quality of work life	• Increase patient access • Increase patient engagement • Extend services provided by organizations adopting a hybrid therapy model	• Help clinicians identify mental illnesses at an earlier stage when interventions may be more effective • Help clinicians customize treatments based on a patient's characteristics
AI-augmentation in MH practice	• Automation technologies could assist alleviate some of the severe staffing shortages in mental healthcare by extending rather than replacing the knowledge and expertise of human clinicians	• AI engagement tools could complement the therapist's interventions in a blended therapy model. To support skill practice between sessions, for example, the therapist could assign a specific GSH component, extending therapy beyond the meeting	• To increase clinicians' readiness to consider using AI tools to help decision-making, there has to be more openness about how algorithms are developed, the data utilized for their creation, and the engagement of mental health practitioners and service users in their evaluation and improvement

AI, artificial intelligence; MH, mental health; GSH, guided self-help.

**Table 2:** Challenges and potential solutions related to pragmatic AI-augmentation.

	**Automation technologies**	**Patient engagement technologies**	**Clinical decision support technologies**
**Potential challenges**			
• Work practices	• Automation complacency	• Required workflow changes	• Lack of interpretability of AI recommendations
• Beliefs and identity	• Fear of obsolescence	• Fear of negative impact on clinician-patient therapeutic alliance	• Concerns about model accuracy and bias • Lack of evidence-based evaluation studies
• Tasks and roles	• Clinician-AI task allocation	• Concerns about patient overreliance on technology	• Clinician surveillance concerns
**Potential solutions**			
• Work practices	• Provide proactive risk assessment • Expose clinicians to potential automation failures	• Engage clinicians in iterative technology development and implementation process	• Train clinicians in computational thinking • Use explainable AI (XAI) techniques
• Beliefs and identity	• Provide more accurate portrayals of capabilities of AI technologies	• Discuss potential risks related to the patient becoming too emotionally attached	• Use human-in-the-loop development • Evaluate efficacy vs. current models
• Tasks and roles	• Add organizational structures and roles to govern AI projects	• Automate detection of threat, and provide recommended action	• Configure AI technologies so that predictions do not infringe upon clinicians' core tasks

AI, artificial intelligence.

## AI-based automation technologies for mental healthcare

### Key technologies for AI-based automation

AI-based automation technologies enable the automation of structured or semi-structured tasks, often *via* computer vision, ML, and NLP. They can perform a wider variety of tasks than traditional automation technologies, such as recognizing conversations and emotions, and therefore can automate or semi-automate certain aspects of care, such as screening, diagnosis, and treatment recommendations, or psychosocial therapy. For example, computer vision in combination with ML offers early autism screening by assessing the eye gaze patterns of children while they watch a short movie on an iPad (11). Platforms using NLP in combination with ML can provide chatbot-based screening as well as assist with routine or mundane tasks that clinicians dislike or are not reimbursed for. For instance, AI-based automation technologies can support measurement-based care by automatically collecting and analyzing data pre-, during, and post-session, administering digital surveys, and generating insights from a patient's talking patterns, tone, and word choice (12).

### Potential benefits of AI-based automation

AI-based automation technologies facilitate the automation of cognitive and physical tasks that are often repetitive and cumbersome, thereby facilitating early screening, increasing patient access, and improving clinician quality of work life. For example, while measurement-based care is perceived to advance MHC and make it more data-informed (13), administering standardized assessments is a laborious task that can be easily computerized. Digital screening tools for autism, eating disorders, or other psychosocial concerns, can be complementary to other assessment approaches and ultimately increase the accuracy, exportability, accessibility, and scalability of screening and intervention (14–16). More generally, these technologies can be used to extend, rather than replace, the skills and experience of frontline clinicians, so could help address some of the acute workforce shortages in MHC (17). This augmented AI methodology could also free up staff time to focus on the human aspects of care. In a blended-care approach, AI-driven chatbots, apps, and online interventions can provide data on a person's responsiveness to them, and quickly identify cases where improvement criteria are not met, prioritizing a higher level of care as needed (18).

### Potential challenges of AI-based automation

With these new potential benefits come new potential challenges. First, regarding clinician daily work practices, these technologies raise the potential for automation complacency. This can occur, for example with an “auto-complete” function for drug names after entering the first few letters. Because clinicians are faced with complex tasks, multitasking, heavier workloads, and increasing time pressures, they may choose the route requiring the least amount of cognitive effort, which may lead to them letting technology direct their path (19). Additionally, clinicians may overestimate the performance of these technologies because they believe that technology has better analytical capabilities than humans, and they might put less effort or responsibility into carrying out a task (20).

Second, regarding beliefs and identities, narratives surrounding AI (21) might influence how frontline clinicians perceive and approach the technology. Because AI-based automation enables the automation of well-defined cognitive tasks that can be captured by sets of patterns and rules, this may lead to clinician concerns about obsolescence. Clinicians may worry that AI will replace the need for their expert work (9, 22).

Third, regarding new tasks, clinicians may be concerned about task allocation between clinicians and AI technologies. They may wonder how the delegation of tasks among interdependent humans and machines should be determined (23). This uncertainty may, in turn, undermine confidence and prevent the widespread use of these technologies.

### Potential solutions for AI-based automation

Regarding potential automation complacency, this issue is not unique to AI technologies, and has been handled effectively in healthcare settings using two strategies. First, before implementing new technology, clinical leaders can first undertake a proactive risk assessment to identify any unexpected vulnerabilities and remediate them (24). Second, leaders can allow clinicians to experience automation failures during training, such as technology failure to issue an important alert or “auto-fill” errors, to encourage critical thinking when using automated systems, and increase the likelihood of recognizing these failures during daily work (25).

Regarding fear of obsolescence, although in the popular imagination software often takes on mythic qualities of near-omnipotence (26), AI will likely augment the importance of human judgement and knowledge (27–29). Clinical leaders could highlight the essential role played by humans at each phase of the development and implementation of an AI technology (30). For example, leaders could note that radiologists may relinquish the preliminary scanning of medical images to the AI technology in order to focus on the more complicated cases, or those that the algorithm finds ambiguous (31); technology developers can include feedback during screening that recommends reaching out for in-person evaluation to verify the clinical recommendation (32).

Regarding concerns about task allocation, the design of AI systems—like the design of other sociotechnical systems—involves decisions about how to integrate augmented intelligence and delegate sub-tasks among humans and non-humans. Clinical leaders at healthcare organizations using AI have put in place a wide range of internal organizational structures and roles to manage and govern AI projects (33). Governance committees should offer a balanced view of challenges and opportunities associated with implementing AI technologies (34).

## AI-enabled engagement technologies for mental healthcare

### Key technologies for AI-enabled engagement

AI-enabled voice-and text-based engagement technologies allow for patient engagement, ranging from the handling of repetitive patient queries to the undertaking of more complex tasks that involve greater interaction, conversation, reasoning, prediction, accuracy, and emotional display. Conversational agents (CAs) such as chatbots use NLP, speech recognition, and synthesis technologies to conduct limited text conversations with human users. Intelligent virtual agents (IVAs) add more realism to conversational emulation through computer-generated, artificially intelligent virtual characters.

IVAs and CAs have been used as coaches to support behavior change such as smoking cessation, starting exercise programs, nutrition, and to provide limited treatment functions, such as elements of cognitive behavioral therapy (CBT), dialectical behavior therapy, or motivational interviewing, in areas where highly scripted protocols are applicable (35). For instance, Woebot is a conversational agent designed to help reduce depression and anxiety. It provides CBT tools, learning materials, and videos as well to guide users through “self-directed” therapy (36). Further, digital guided self-help (GSH) programs can improve access to evidence-based therapy and offer patients standardized interventions (37).

### Potential benefits of AI-enabled engagement

While these technologies cannot function at the level of an experienced, empathic human clinician, they may play a special role in MHC prevention and intervention, both supportive of and distinct from the functions of human healthcare providers. AI-enabled engagement technologies have the potential to increase patient access because they offer 24/7 availability in remote areas where access to MH professionals may be limited (38). They can also increase the dissemination of evidence-based practices. For instance, “Elizabeth,” a virtual nurse, assists patients in understanding information about hospital discharge, such as follow-up care and prescription needs (38). AI can also identify program participants' disengagement and alert the assigned case manager or clinician, using hundreds of datapoints that humans do not always have access to or cannot process in a timely manner (39).

In addition, these technologies may provide between-session support through facilitating homework and skills practice. In a blended therapy model, AI tools for engagement augment clinicians' interventions. Similarly, AI-augmented engagement can extend the services provided by organizations adopting a hybrid therapy model—integrating both in-person and technology-based modalities (40). For instance, clinicians can assign a specific GSH module, thereby extending therapy beyond the meeting (37). Further, a study on the use of CAs in inner city outpatient clinics found that patients with chronic pain and depression who interacted with a CA reported a high degree of compliance with the CA's recommendations for stress reduction and healthy eating (37).

### Potential challenges of AI-enabled engagement

Just as AI-enabled engagement technologies may afford new benefits for MH clinicians, so they may raise a new set of new challenges. First, regarding clinician daily work practices, these technologies will require workflow changes that enable clinicians to collaborate with chatbots and patients, and there are no codified practices that clinicians can use to implement chatbots in a way that meets patients' needs, goals, and lifestyles while facilitating trust in AI to improve MH outcomes (41).

Second, regarding beliefs and identities associated with AI-Enabled Engagement, recent research on CAs and GSH have found that program participants report forming some sort of a therapeutic alliance with automated MH platforms. For instance, the users of a smoking cessation chatbot reported that their perceived therapeutic alliance with the chatbot increased over time (42). Patients forming a relationship with the chatbot may lead to clinician concerns about a negative impact on the therapeutic alliance between clinicians and patients (43). Further, the literature has noted that the therapeutic alliance with a human being and with a therapist may be distinct concepts that need to be assessed using different tools (44).

Third, regarding concerns about task allocation, clinicians may be concerned that their patients may assume that the technology is adequately addressing their MH needs, and may over-rely on technology (7). Additionally, there is a potential risk of harm to patients if a system is unable to appropriately address circumstances in which a user needs rapid crisis care or if other safety-related action is necessary (43). Risk assessment is complicated enough for providers; if we delegate it entirely to machines, patients' unique circumstances may be overlooked (45–47).

### Potential solutions for AI-enabled engagement

Regarding workflow changes required by AI-enabled engagement technologies, research on AI technology implementation in other healthcare domains has shown that clinical leaders can facilitate the creation of new workflows by supporting an iterative process between AI technology's developers and clinicians—through which both the clinicians' daily activities and the technology itself are transformed (48).

Regarding a potential negative impact on the therapeutic alliance, clinical leaders could encourage the responsible party to discuss with the patient any potential risks related to the patient relying on the agent more than on human caregiver or clinician. Technology developers could design the technology to detect if the user is becoming too emotionally dependent, broach this topic with them, and discuss possible risks (7).

Third, regarding clinician concerns about task allocation, technology developers should include an explicit message to the patient outlining circumstances under which patients should seek clinician help. There is a need for blending AI insights with clinician-led engagement to incorporate the nuances, risk, and support resources an individual has. Developers could design agents to identify types of risks and take appropriate action, such as detecting suicide risk and immediately directing the patient to contact a suicide prevention hotline, a clinician, or caregiver (7).

## AI-enabled decision support technologies for mental healthcare

### Key technologies for AI-enabled decision support

AI-enabled decision support technologies use ML algorithms to make predictions based on past data. New large-scale advances, such as deep or reinforcement learning (49, 50), have enabled AI to make inroads into much more complex decision-making settings, including those that involve audio and speech recognition. These improved decision-making processes stem from big datasets now available in healthcare, that are enhanced by computational analyses (51). For example, supervised ML has been shown to predict attention-deficit and hyperactivity disorder (ADHD), autism, schizophrenia, and Tourette syndrome and suicidal ideation. ML-based models have been shown to more accurately predict the severity of depression or anxiety (52), and stroke (53) and allow clinicians to identify which interventions will be most effective for which patient populations.

### Potential benefits of AI-enabled decision support

AI-enabled decision support technologies can enable clinicians to detect mental illnesses at an earlier stage when interventions may be more effective, and offer customized treatments (1). These technologies, as opposed to clinicians who are human, never grow weary, can integrate new knowledge into their models, and can process large datasets in a matter of minutes. However, algorithms do not have the empathy and capacity for nuance of a human being. Therefore, platforms that integrate data from multiple resources such as assessments, the treatment alliance, content from treatment sessions, and the patient's perception of therapy, are poised to augment, rather than replace, clinician decision making (54).

### Potential challenges of AI-enabled decision support

With these new potential benefits comes a set of new challenges. First, regarding clinician daily work practices, ML model outputs are often complex and uninterpretable to humans—the so called ‘black box' problem. The precise reason for an ML model's recommendation often cannot be easily pinpointed, even by data scientists (31). In addition, technology-based approaches are pattern-based, while traditional research methods are hypothesis-driven, and most clinicians do not have the training required to engage in the computational thinking associated with these interpretations (17, 37).

Second, regarding beliefs and identities, clinicians may not trust that ML-based algorithms yield accurate predictions and that recommendations can be integrated into clinical practice securely and efficiently for the benefit of patients. There is currently a low quality and quantity of evaluated evidence-based studies for these technologies (55). In the healthcare context, lives may depend on assuring that model predictions not only are accurate in the original context, but also function robustly in other contexts, and that the results of predictive models are not biased (17).

Third, regarding concerns about task allocation, AI decision-support tools are by their nature prescriptive in that they recommend actions for the clinician to take. Clinicians may be concerned that the availability of AI-enabled decision support technologies may allow others from outside their domain to attempt to scrutinize and dictate frontline clinicians' diagnosis and treatment decisions (56).

Fourth, most studies on AI-augmentation for personalizing therapy have been carried out on clients with depression and anxiety (57). AI-based clinical decision-support technologies have been understudied in severe mental illnesses (SMI), including schizophrenia, bipolar disorder, severe depressive disorder and psychotic disorders (58).

### Potential solutions for AI-enabled decision support

To address the black box problem and misperceptions of ML models, clinicians should be informed about the advantages, limitations, and risks of AI-based systems. Such knowledge and abilities are required so that clinicians feel confident in using AI and communicating the results effectively with patients and carers. The more technology developers incorporate explainable Artificial Intelligence (XAI), i.e., explanatory techniques that clearly state why the AI has made a specific suggestion, the more they can increase the credibility, accountability, and trust in AI in mission-critical fields like MHC (59). Developers should also consider the audience when determining what explanations to provide; for example, clinicians may be most interested in explanations that make the model's functioning easy to understand, and in those that help them trust the model itself (60).

Second, regarding therapists' concerns about how AI-enabled decision support technologies can be incorporated effectively and safely into clinical practice, developers need to foster greater involvement of MH providers and service users in AI models evaluation and refinement. Developers can use a human-in-the-loop approach to data collection and model creation that enlists MH practitioners in all stages of development and use (61). There also needs to be more separation between studies that measure degree of use of AI technologies, and those that evaluate the efficacy of these technologies; in order to gain the trust and understanding of clinicians, developers need to demonstrate that these technologies work better than existing service delivery (17, 62). For example, in the case of stroke risk prediction and prevention, this means that novel ML-based approaches need to compete against established models to win clinicians' and patients' trust (53).

Third, regarding task allocation, research on AI technology implementation in other healthcare domains has shown that clinician concerns regarding autonomy and surveillance can be addressed by clinical leaders facilitating greater clinician involvement in developing these technologies and by developers configuring the AI tools so that their predictions do not infringe upon clinicians' core tasks, but instead assist them with important tasks less valued by the clinicians such as monitoring the completion of follow-up items (56).

Fourth, in regard to the claim that AI-based decision support tools should be expanded to SMI, since these conditions may be relatively complicated to diagnose, we suggest that efforts to develop technologies for AI decision support adopt human-in-the-loop methods to develop, assess, refine, and test the tools before they are deployed in practice. In addition, technology developers should give more attention to the impacts of using these tools to tailor and personalize treatment (58).

## Conclusion

While much has been written about the potential for AI technologies to transform the delivery of MHC, the predominant focus to date has been on the technical, ethical, and regulatory challenges associated with these technologies. What is missing is a pragmatic approach to AI-augmentation that outlines challenges and potential solutions related to the incorporation of AI-based technologies into clinicians' everyday work. Such an approach can help frontline clinicians use AI-based technologies to transform how MH is understood, accessed, treated, and integrated, as clinicians make decisions, day in and day out, to care for their patients in need.

## Data availability statement

The original contributions presented in the study are included in the article/supplementary material, further inquiries can be directed to the corresponding author.

## Author contributions

KK and SS-S: conceptualization and writing—review and editing. All authors contributed to the article and approved the submitted version.

## Conflict of interest

Author SS-S is the Chief Clinical Officer of Eleos Health. The remaining author declares that the research was conducted in the absence of any commercial or financial relationships that could be construed as a potential conflict of interest.

## Publisher's note

All claims expressed in this article are solely those of the authors and do not necessarily represent those of their affiliated organizations, or those of the publisher, the editors and the reviewers. Any product that may be evaluated in this article, or claim that may be made by its manufacturer, is not guaranteed or endorsed by the publisher.
